# Dural arteriovenous fistula associated with medullary ependymoma: an unusual association

**DOI:** 10.31744/einstein_journal/2024AI0998

**Published:** 2024-10-23

**Authors:** Benedito Jamilson Araújo Pereira

**Affiliations:** 1 Universidade de São Paulo Faculdade de Medicina Department of Neurology São Paulo SP Brazil Laboratory of Molecular and Cellular Biology (LIM15), Department of Neurology, Faculdade de Medicina, Universidade de São Paulo, São Paulo, SP, Brazil.

**Figure 1:**
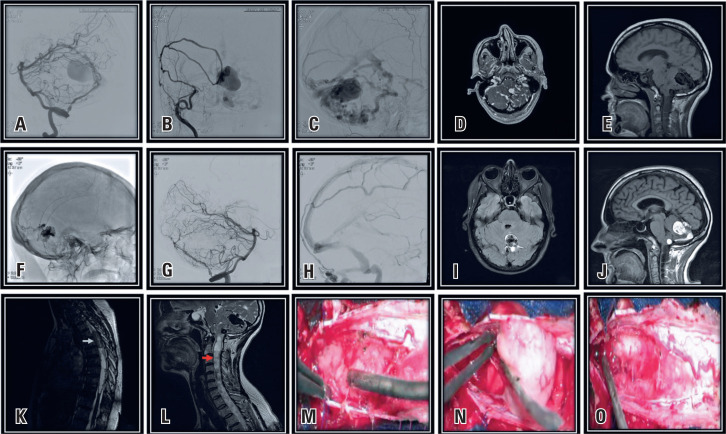
A-C) Cerebral angiography showing the presence of high-flow dural arteriovenous fistula (DAVF), along with venous aneurysm, being nourished by the superficial temporal and occipital arteries and middle pharyngeal branches; D-E) Cerebral MRI showing DAVF in posterior fossa; F-H) Cerebral angiography after endovascular treatment of DAVF; I-J) MRI after endovascular treatment with Onyx; K) Thoracic spine MRI showing intramedullary tumor; L) MRI of cervical spine showing syringomyelia extending from medullary tumor to medulla; M-O) Transoperative images of spinal tumor resection

A 42-year-old woman presented with progressive paraparesis; after magnetic resonance imaging of the neuroaxis, an intramedullary tumor was observed at T4-T6 with a syringomyelia and dilatation of the venous system of the posterior fossa. Cerebral angiography revealed dural arteriovenous fistula (DAVF). To treat DAVF, the medullary tumor was surgically resected, and histopathological analysis confirmed the presence of an ependymoma (Figure 1). The presence of DAVF associated with spinal tumors has not been reported in the literature to date, which makes this article unique.

Ependymomas are highly vascular. Tumor-induced biochemicals such as a vascularization factor, hypoxia-inducible factor 1, vascular endothelial growth factor, and other thrombotic chains might contribute to the generation of DAVF.^(1,2)^

Another hypothesis is that changes occur in cerebrospinal fluid (CSF). CSF alterations that lead to intracranial hypertension are generally those that obstruct cerebrospinal fluid circulation at any point in its pathway and those that cause difficulty in CSF reabsorption.^(3)^

Benign tumors may be associated with DAVFs that are directly related to the cancer or are present in distant anatomical locations. This case report such an association between DAVF and medullary ependymoma.
